# Heat‐Driven Iontronic Nanotransistors

**DOI:** 10.1002/advs.202204120

**Published:** 2023-01-25

**Authors:** Domenic Prete, Alessia Colosimo, Valeria Demontis, Luca Medda, Valentina Zannier, Luca Bellucci, Valentina Tozzini, Lucia Sorba, Fabio Beltram, Dario Pisignano, Francesco Rossella

**Affiliations:** ^1^ NEST Scuola Normale Superiore and Istituto Nanoscienze‐CNR Piazza San Silvestro 12 Pisa I‐56127 Italy; ^2^ Universitá di Pisa Dipartimento di Fisica Largo Bruno Pontecorvo, 3 Pisa 56127 Italy; ^3^ Scuola di Ingegneria | Dipartimento di Scienze Fisiche Informatiche e Matematiche Universitá di Modena e Reggio Emilia via Campi 213/a Modena 41125 Italy

**Keywords:** iontronics, nanoelectronics, nanowires, polyelectrolytes, thermoelectric

## Abstract

Thermoelectric polyelectrolytes are emerging as ideal material platform for self‐powered bio‐compatible electronic devices and sensors. However, despite the nanoscale nature of the ionic thermodiffusion processes underlying thermoelectric efficiency boost in polyelectrolytes, to date no evidence for direct probing of ionic diffusion on its relevant length and time scale has been reported. This gap is bridged by developing heat‐driven hybrid nanotransistors based on InAs nanowires embedded in thermally biased Na^+^‐functionalized (poly)ethyleneoxide, where the semiconducting nanostructure acts as a nanoscale probe sensitive to the local arrangement of the ionic species. The impact of ionic thermoelectric gating on the nanodevice electrical response is addressed, investigating the effect of device architecture, bias configuration and frequency of the heat stimulus, and inferring optimal conditions for the heat‐driven nanotransistor operation. Microscopic quantities of the polyelectrolyte such as the ionic diffusion coefficient are extracted from the analysis of hysteretic behaviors rising in the nanodevices. The reported experimental platform enables simultaneously the ionic thermodiffusion and nanoscale resolution, providing a framework for direct estimation of polyelectrolytes microscopic parameters. This may open new routes for heat‐driven nanoelectronic applications and boost the rational design of next‐generation polymer‐based thermoelectric materials.

## Graphical abstract

A novel class of thermally‐driven, hybrid nanotransistors based on InAs nanowires is developed. Semiconductor nanostructure devices are embedded in Na^+^‐functionalized (poly)ethyleneoxide and allow for extracting the parameters featuring ionic thermodiffusion. Reported results open new perspectives for heat‐driven nanoelectronic applications and boost the rational design of next‐generation polymer‐based thermoelectric materials.

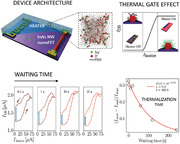

## 1 Introduction

Architectures and technologies at the nanoscale might have a huge impact on how heat and heat transport are conceived and employed,^[^ ^1^, ^2^, ^3^ ^]^ providing accurate tools for the optimal exploitation of thermal budgets^[^ ^4^, ^5^ ^]^ and boosting the development of novel thermoelectric materials.^[^ ^6^, ^7^, ^8^ ^]^ Among these, polyelectrolytes^[^ ^9^ ^]^ are emerging as ideal flexible^[^ ^10^, ^11^ ^]^ and bio‐compatible^[^ ^12^ ^]^ systems. In fact they were proposed for self‐powered implantable and wearable devices,^[^ ^13^, ^14^, ^15^ ^]^ sensors^[^ ^16^ ^]^ and energy harvesters.^[^ ^17^, ^18^, ^19^ ^]^ In ionic thermoelectric devices exploiting polyelectrolytes, energy harvesting can be achieved by charging supercapacitors^[^ ^20^, ^21^ ^]^ or by deploying field effect to control the electrical conductivity of semiconductors through electric double layers.^[^ ^22^ ^]^ Due to the small length scale of the double layer (≈1 nm), the latter method is very well suited for driving nano‐sized semiconductor systems, whose high surface‐to‐volume ratio ensures strong coupling for electrolyte gating. Semiconducting nanowires were demonstrated to be highly appropriate to this aim.^[^ ^23^, ^24^, ^25^, ^26^ ^]^ Conversely, their reduced lateral dimension may allow them to probe the local ionic density with high spatial resolution thus yielding access to microscopic parameters of interest for the design of novel thermoelectric polyelectrolytes and for material characterization.

Surprisingly, while the unique features of polyelectrolytes are known to arise from ionic dynamics occurring on the nanometer scale, to date the implementation of a nanodevice capable of probing the behavior of these materials on the relevant space and time scales is still missing. We bridge this gap by combining an InAs nanowire with Na‐functionalized (poly)ethylene oxide (PEO), and by exploiting the Soret effect^[^ ^27^ ^]^ to drive field‐effect modulation of the semiconductor electrical conductivity. Using the nanowire as a nanoscale probe for ion dynamics and resorting to multiscale molecular dynamics simulations and finite element analysis, we relate the operational regimes and hysteretic behavior of this class of devices, providing a new platform to evaluate microscopic parameters of the polyelectrolyte and enabling for the first time the direct quantitative estimate of the ionic thermalization time with a nanodevice.

To demonstrate single nanowire‐based, planar electronic nanodevices driven by means of ionic thermodiffusion in polyelectrolytes, we embedded InAs nanowires in a droplet of functionalized PEO, with Na^+^ ions being able to diffuse in the polymeric matrix composed by neutral PEO and by charged (deprotonized) PEO‐ chains. Temperature gradients are applied by means of metallic heating elements fed by an AC current, ensuring that the ionic diffusion is caused by heating effects rather than the establishment of electric field gradients. This is assured by the low electrical resistance of the metallic heating element, which results in very low potential drops between the heater and the nanowire, unable to drive any ionic diffusion by merely electrostatic effects. The resulting thermodiffusion‐driven field effect on the nanowire is investigated by analyzing the influence of heat source distance, bias current amplitude, frequency and thermalization time on device operation as well as on its hysteretic behavior. Experimental data are combined with molecular dynamics simulations and finite element analysis to link our observations with microscopic polyelectrolyte quantities that are critically important for the operation of nanoscale heat‐driven iontronics.^[^ ^28^ ^]^ Our approach may provide new directions for the rational material design of polyelectrolytes for thermoelectric conversion, driving the development of nanodevices for local temperature sensing, heat‐driven electric memories, and other electronic components.

## 2 Results and Discussions

### 2.1 Heat‐Driven Iontronic Transitors at the Nanoscale: Device Architecture and Operational Principles

**Figure 1:**
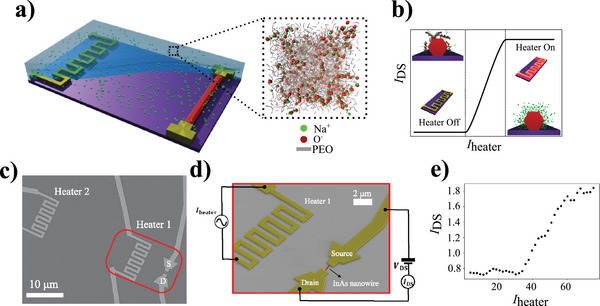
Heat driven iontronic nanotransistor architecture and operation. a) Device pictorial view. A metallic serpentine acting as heater and an electrically contacted InAs nanowire are embedded into a polyelectrolyte. Inset: simulated molecular distribution of the polyelectrolyte at thermodynamic equilibrium. b) Current amplitude, *I*_DS_, in the nanowire versus heater current amplitude, *I*_heater_:expected behavior. Large *I*_heater_ generates a temperature gradient driving net accumulation of Na^+^ ions at the polyelectrolyte/nanowire interface, yielding field effect modulation of *I*_DS_. c) Scanning electron micro‐graph of a prototypical device. Two heaters are positioned 7 and 35 µm away from the nanowire. d) Tilted scanning electron micrograph zoomed on the nanowire. The circuital scheme of the experimental setup, in overlay, shows the AC current generator feeding the heater, the voltage source and ampere‐meter for the measurement of the nanowire current. e) Typical heat‐driven device operation (*I*_heater_ frequency 72 Hz, waiting time 120 s). *I*_heater_ is found to drive a modulation of *I*_DS_ reproducing the main features of the curve reported in panel (b). Here the maximum temperature difference, $\Delta T_{\max}$, between heater and nanowire is 0.6 K, as result from finite element analysis (Section S3, Supporting Information).

The developed device architecture for the demonstration of our thermodiffusion‐driven ion‐gated transistor operation is reported in **Figure** **1**. Prior to current injection in the heating element, the charged species are uniformly distributed in the bulk of the droplet, as shown in the pictorial representation in Figure 1a. The blow‐up in Figure 1a schematically displays the distribution of the polymer chains (grey = neutral, red = dissociated) and Na^+^ ions (green dots) as obtained by combining atomistic and coarse grained molecular dynamics simulations (see the Section S1, Supporting Information and supplementary movies for more detail). When an AC current biases the heater, a temperature gradient is established in the polyelectrolyte and the ionic species start to diffuse, resulting in the accumulation of Na^+^ ions near the nanowire surface and consequently in the modulation of the charge carrier density—hence the current—in the nanowire via the electric double layer gating mechanism (Figure 1b). Further details of the devices are presented in Figure 1c,d. The measurement reported in Figure 1e shows a typical thermoelectric gating operation and is consistent with the expected behavior schematically reported in Figure 1b. Here, the field effect modulation of the drain to source current in the nanowire reported in Figure 1e results in an on–off ratio of the current of a factor ≈2. This relatively small modulation is connected to the ionic concentration achievable locally at the electrolyte/nanowire interface as a result of the heat‐driven ionic diffusion, combined with the high doping level of the used semiconductor nanostructure. Possible spurious effects related to charged chemical impurities in the polyelectrolyte and unwanted substrate heating were ruled out, as discussed in Section S2, Supporting Information. It is worth stressing here that the exploited mechanism for the achievement of the field effect control over the semiconductor conductance is rather different when compared with conventional electrostatic gating techniques. Specifically, in the conventional ion‐gating operation, a DC voltage is applied to a metallic counter‐electrode which in turn drives an electric field controlling the arrangement of ions in the electrolyte. This operation can lead to electrochemical reactions and unwanted leakage currents in the electrolyte if the applied voltage exceeds the electrolyte electrochemical window. On the opposite hand, in our device the ions are driven by thermal gradients rather than by electric field gradients, which are negligible thanks to the reduced heater surface and the current‐heating mechanism, combined with the metallic serpentine low electrical resistance: thus, overall the system is safely protected by any leakage currents of electrochemical nature.

In the following, we investigate functional parameters of this architecture and analyze the system response under several different combinations of heater distance, AC current frequency and measurement waiting time—that is, the time interval passing between the application of I_heater_ and the measurement of *I*_DS_. We shall address the relation between these parameters and the dynamics of the thermal and electrical response of the polyelectrolyte. We shall argue that this result demonstrates the potential of this technology for a new generation of heat‐driven iontronic nanodevices.

### 2.2 Impact of the Heater‐Nanowire Distance on the Iontronic Nanotransistor Operation

**Figure 2:**
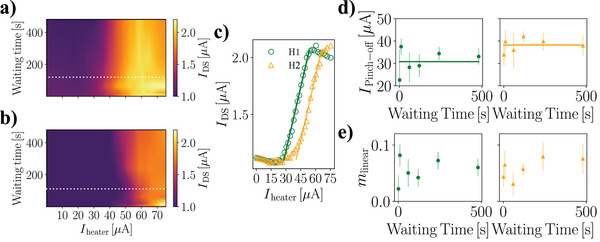
Impact of heater distance on heat driven nanotransistor operation. a,b) Drain–source current *I*_DS_ measured with respect to the amplitude of the heating current *I*_heater_ and the waiting time, employing Heater 1 and Heater 2. AC frequency is 72 Hz. c) $I_{\mathrm{DS}}\text{--}I_{\mathrm{heater}}$ characteristics extracted from dataset reported in (a) and (b). The waiting time of the reported curves is 120 s, corresponding to the white dashed lines in panels (a) and (b). d) Analysis of the pinch‐off current $I_{\mathrm{Pinch}-\mathrm{off}}$ for several values of waiting time. Different mean values for $I_{\mathrm{Pinch}-\mathrm{off}}$ are found for the operation with the two heaters. Specifically, $I_{\mathrm{Pinch}-\mathrm{off},\;\mathrm{Heater}1}=30\pm 3\;\mu A$ and $I_{\mathrm{Pinch}-\mathrm{off},\;\mathrm{Heater}2}=40\pm 5\;\mu A$. e) Analysis of the transconductance for different waiting times, evaluated as the slope of the $I_{\mathrm{DS}}\text{--}I_{\mathrm{heater}}$ characteristics in the linear regime. The values for *m*_linear_ are comparable within the experimental uncertainty for device operation with both heaters for each value of waiting time.

In order to probe ionic diffusion at the nano/micro scale with the developed architecture, we first investigate the impact of the distance between the heating element and the nanowire sensor on device operation by comparing the measurements performed with heaters located at different distances from the nanowire. **Figure** **2**a,b report the drain‐source current, *I*_DS_ (false color, see color coding on the right panels), measured upon activating either the “Heater 1” or the “Heater 2” imaged in Figure 1c, for different waiting times. The white dashed lines correspond to the *I*_DS_ plotted in Figure 2c and allow a direct comparison of $I_{\mathrm{DS}}\text{--}I_{\mathrm{heater}}$ characteristics after 120 s waiting time for the two configurations. The operation with the two heaters leads to a noticeable difference between the pinch‐off values for the heating current $I_{\mathrm{Pinch}-\mathrm{off}}$: the farther the heater, the more *I*_heater_ current is needed to enter the linear regime. This behavior is compatible with the larger spatial scale involved in the set‐up of the temperature gradient, corresponding to a higher power to be dissipated in the heating element in order to achieve a comparable effect with respect to the nearer heater. The entity of this shift is confirmed by the plots in Figure 2d, reporting the values for $I_{\mathrm{Pinch}-\mathrm{off}}$ for several waiting time values in thermoelectric gating performed with Heater 1 or Heater 2. The mean values for the measured pinch‐off currents, $I_{\mathrm{Pinch}-\mathrm{off}}$, are 30 ± 3 µA for Heater 1 and 40 ± 5 µA for Heater 2. We also analyzed another parameter signature of the thermoelectric gating performance: the transconductance *m*_linear_, evaluated as the slope of the linear regime in the $I_{\mathrm{DS}}\text{--}I_{\mathrm{heater}}$ characteristics. Figure 2e shows the transconductance values for several waiting times, measured for device operation with either Heater 1 or Heater 2. The *m*_linear_ values estimated by operating the device with both heaters are found to be comparable within experimental uncertainty, implying that the heat source‐nanowire distance does not affect the thermoelectric gating mechanism performance, rather it causes a rigid shift of the features observed in the $I_{\mathrm{DS}}\text{--}I_{\mathrm{heater}}$ characteristics.

### 2.3 Impact of the Heating Input Frequency on the Response of Iontronic Nanotransistors

**Figure 3:**
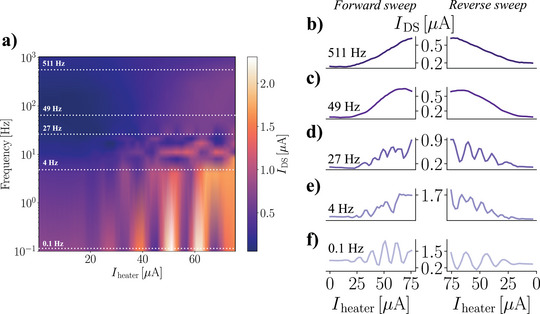
Frequency dependence of heat driven nanotransistor. a) Measured *I*_DS_ dependence on injected heater current amplitude and frequency. An oscillatory behavior for f<50Hz is evident. b–f) $I_{\mathrm{DS}}\text{--}I_{\mathrm{heater}}$ curves corresponding to white dashed lines visible in panel (a), measured sweeping the heating current from 0 to 75 µA (Forward sweeps) and from 75 to 0 µA (reverse sweep). In panels (b) and (c), corresponding to high frequency measurements (f>50Hz) the observed ion gating effect is smooth and monotonic, while in panels (d–f) oscillations in the measured *I*_DS_ are visible, corresponding to an unstable heating configuration, in which the ionic dynamic is faster than the external heat pulses driving ionic thermo‐diffusion.

We further investigate the *I*_DS_ dependence on the heating current amplitude and frequency (**Figure** **3**). An oscillatory behavior of *I*_DS_ versus *I*_heater_ is observed for frequencies in the range 0–48 Hz, whereas for *f* exceeding 50 Hz a smooth monotonically increasing behavior is observed (Figure 3a). The differences between these two frequency regimes are further highlighted in Figure 3b–f, in which several horizontal cuts of the color‐plot reported in Figure 3a are reported. Indeed, if the oscillation period of the electrical signal responsible for the establishment of the temperature gradient is of the same order of (or longer than) the ionic diffusion time, the ions may not reach an equilibrium state, due to scattering events causing them to counter‐diffuse against the temperature gradient. On the opposite side, when the period of the heating current oscillations is reduced, the ions are not able to react to the temporal variation of the heating source and feel a constant ‘mean’ temperature gradient which uniformly drives them away from the heat source. The occurrence of these two regimes is ascribable to the coexistence of two different time scales in the hybrid semiconductor/polyelectrolyte device architecture, the first being the (slow) timescale featured by the ionic transport properties of the polyelectrolyte, while the second (fast, i.e., instantaneous with respect to the previous one) is related to the electronic response of the nanowire. The latter allows to probe the variation of the ionic landscape surrounding the nanostructure within time scales much smaller than the characteristic times needed for the ions to diffuse. Ultimately, this enables the employment of the developed architecture to measure a key parameter of the polyelectrolyte, namely the ionic thermalization time, as shown in the following.

### 2.4 Device Hysteretic Behavior and Ionic Thermalization Time

**Figure 4:**
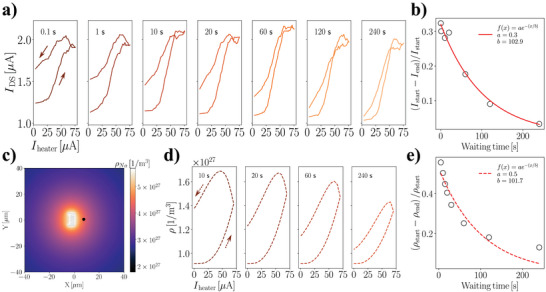
Hysteresis analysis. a) $I_{\mathrm{DS}}\text{--}I_{\mathrm{heater}}$ characteristics measured by operating the device with Heater 1, *f* = 72 Hz. Each curve corresponds to a different waiting time, and acquisitions are performed by sweeping *I*_heater_ forward and backward in order to appreciate any hysteresis in the measured *I*_DS_. b) The hysteresis parameter $\left(I_{\mathrm{start}}-I_{\mathrm{end}}\right)/I_{\mathrm{start}}$ is extracted for each measured curve. An exponential fit of the experimental points was performed, $f\left(x\right)={\text{ae}}^{-x/b}$. Fit results return a=0.3±0.01 and b=102.9±11.5s. c) Result of a finite element analysis simulation showing the radial distribution of the Na^+^ ions in response to the applied temperature bias when $I_{\mathrm{heater}}=75\;\mu A$ and waiting time is 240 s. d) Calculated $\rho \text{--}I_{\mathrm{heater}}$ curves for several values of waiting time, ρ being the net positive charge density at the point highlighted with the black dot in panel (c). The overall hysteretic behavior of the computed curves is consistent with experimental observations. e) The hysteresis parameter for the calculated curves is extracted as (ρ_start_ − ρ_end_)/ρ_start_ and follows an exponentially decaying behavior. In this case a fitting procedure returns a=0.5±0.04 and b=101.7±15.5s, in agreement with the experimental counterparts.

**Figure** **4**a shows the device response against several different waiting times ranging from 0.1 to 240 s. Curves measured with waiting times (≲60 s) show pronounced hysteresis, indicating the occurrence of a heat‐driven memory effect which may be thought to be exploited to implement volatile memory functionalities on this device architecture owing to the inherently volatile nature of the thermodiffusion mechanisms underlying the observed behavior. On the contrary, the hysteresis is firmly reduced in the curves measured with waiting time ≳120 s, suggesting that the ionic configuration is thermalized in the applied temperature gradient. We extract the hysteresis parameter by calculating the difference between *I*_DS_ values at the beginning and the end of each *I*_heater_ sweep and normalizing on the value of I_DS_ at the beginning of the acquisition. This parameter, $\alpha =\left(I_{\mathrm{start}}\text{--}I_{\mathrm{end}}\right)/I_{\mathrm{start}}$, is plotted with respect to measurement waiting time in Figure 4b, clearly showing an exponentially decaying behavior (a fit with the function *f*(*x*) = *ae* ^−*x*/*b*^ returns: a=0.3±0.01, b=102.9±11.5s). The b parameter can be assumed to be linked to the time interval needed by the ions to reach an equilibrium state in presence of the temperature gradient, that is, their thermalization time.

The observed hysteretic features are reckoned to derive from the heat‐driven mass transport dynamics of the polyelectrolyte. In order to validate this scenario, we perform finite element modeling (for details, see Section S3, Supporting Information), computing the charge spatial density distribution ρ in the polyelectrolyte, as reported in Figure 4c–e. The equations describing mass transport rely on the diffusion coefficients of the species composing the mixture: these are computed resorting to both atomistic MD simulations of a three component mixture (undissociated PEO chains, dissociated charged PEO and Na^+^, with a dissociation ratio of 1/2) and to coarse grained simulations at the monomer level resolution (Section S1, Supporting Information). Figure 4d displays net positive charge density profiles at the location of the nanowire (i.e., at a point 7 µm away from the heater, corresponding to the black dot in Figure 4c) for several values for waiting times: the hysteretic behavior is reproduced by the computed curves, indicating that the observed hysteretic features are linked to the mass transport dynamics of the polyelectrolyte. We extract the hysteresis parameter by computing (ρ_start_ − ρ_end_)/ρ_start_, which confirms the exponentially decaying behavior of the hysteresis as shown in Figure 4d. The fitting procedure reported in Figure 4e returns a=0.5±0.04 and b=101.7±15.5s. These values are coherent with the those extracted from the experimental curves, indicating that the microscopic parameters used in the simulations allow to reconstruct the experimentally observed phenomenology, resulting in high agreement within the values for microscopic parameters computed by the simulations and extracted by experimental datasets. It is worthwhile to mention here that these values rely on the extraction of the microscopic parameters locally and takes into account any effect coming from local temperature variations; this is not the case for different measurement techniques that in principle could provide access to these parameters, which rely on non‐local measurements and thus are less sensitive to local effects compared to the architecture developed in this work. While we are not aware of experimental investigations which use conventional techniques to estimate diffusion and thermalization time of the system used in our work, however, we consider that the combined use of dielectric spectroscopy, fast differential scanning calorimetry and specific heat measurement setup might be a good strategy to reach the goal. Notably, this protocol can be employed to evaluate microscopic parameters of any polyelectrolyte by fitting α, providing a novel experimental tool for the facile assessment of the ionic diffusion coefficient.

## 3 Conclusions

We have demonstrated the use of Na‐functionalized PEO as heat driven gate of InAs nanowire transistors, harnessing the nanostructure as a nanoscale probe of the polyelectrolyte ion dynamics. Combining state‐of‐the‐art semiconductor nanomaterials and nanodevice fabrication techniques with finite element modeling and molecular dynamics tools, we have related the field effect, resulting from the ionic thermo‐diffusion, to the microscopic charge transport dynamics of the ionic thermoelectric material. This allowed us to provide the quantitative estimation of the ionic thermalization time, directly extracted from the analysis of the device response. Interestingly, one could envision different routes for testing the thermally‐driven operation of the device concept developed in our work. A possible strategy might be exploiting a light beam in order to—simultaneously—heat the polymer and measure the local temperature of polymer and substrate, for instance using micro‐Raman spectroscopy. Overall, taking this challenge could represent the ambitious, medium‐term perspective of the present work. The reported functionalities of the nanodevices may by employed for sensing continuous and intermittent temperature gradients at the microscale. Moreover, we have identified two distinct functional regimes related to the charge and mass transport dynamics of the polyelectrolyte: a high‐hysteresis regime with potential applications for thermally‐activated memories, and a low‐hysteresis regime, which is ideal for heat driven nanoelectronic operation. The developed approach might be applied in principle to any semiconductor/polyelectrolyte combination and might take a huge boost from the exploitation of patterning techniques allowing to achieve scalable flexible device production. Furthermore, the operation of the semiconducting nanowire as a probe for the local ionic density of the polyelectrolyte might be optimized with a suitable choice of the material electrical properties, enhancing the semiconductor electrical switching response and thus the sensitivity to local charge distribution changes. To this regard, an electron doping level of the semiconductor nanostructure below 10^16^ cm^−3^ should likely yield to off‐on ratio enhancement of an order of magnitude, passing from 2 to 20 or above. Our work shines new light on ionic diffusion phenomena at the nanoscale, and will be relevant for the design of flexible thermally‐driven nanoelectronics, as well as efficient energy harvesters for self‐powered devices.

## 4 Experimental Section

### Nanowire Growth

InAs nanowires (n‐type, $n\approx \;5\times \;10^{17}{\mathrm{cm}}^{-3}$) were grown by chemical beam epitaxy (CBE) in a Riber Compact‐21 system on InAs (111)B substrates by the Au‐assisted growth. A thin (0.5 nm) Au film was deposited on the substrates at room temperature by thermal evaporation in a metallization chamber equipped with quartz crystal thickness monitor. The substrate was then introduced in the CBE chamber and a thermal annealing (20 min at 450 ± 10 °C under As flux) was carried out to trigger Au dewetting and NPs formation. After lowering the temperature to 410 ± 10 °C, the nanowire growth was started. Trimethylindium and tert‐butylarsine were used as metalorganic precursors, with line pressures of 0.6 and 1.5 Torr, respectively. Se obtained from ditertiarybutylselenide precursor was used as dopant at fixed line pressure of 0.2 Torr. After 40 min the growth was stopped, and the sample cooled down to 150 °C under As flux. Morphological characterization of the grown NWs was performed using a Zeiss field‐emission SEM operated at 5 kV. The average diameter and length of the nanowires were 45 ± 10 nm and 1500 ± 30 nm, respectively.

### Device Fabrication

As grown were detached by the growth substrate via sonication in isopropyl alcohol (IPA). A droplet of IPA with nanowires in suspension was casted on the fabrication substrate on which bonding pads were previously fabricated by means of UV lithographic techniques. Devices were fabricated by means of electron beam lithography, using a layer of PMMA‐based resist. After lithography nanowire surface was passivated by means of a conventional ${\left({\mathrm{NH}}_{4}\right)}_{2}S_{X}$ solution in order to guarantee good ohmic contacts. Subsequently, a Ti/Au bilayer (10/100 nm) was thermally evaporated. After a standard lift‐off procedure aided by dipping the samples in hot acetone for 5 min, devices were wire bonded on commercial dual‐inline packages and a droplet (⩽0.1 µL) of the polyelectrolyte was applied prior to measurements.

### Polyelectrolyte Synthesis

PEO (400 g mol^−1^) was stored in a three neck round flask and dehydrated overnight by means of a vacuum pump. In order to remove the terminal hydrogen from hydroxyl groups of the PEO chains a pellet of metallic sodium (6% w/w Na/PEO ratio) was added to the flask, stirred at 30 rpm and kept at 35 °C by means of a water bath. After 24 h the PEO was desiccated and then used for experiments.

### Measurement Setup

Electrical transport measurement of semiconducting nanowires were performed with a Keithley 2600 dual‐channel SourceMeter. A DC bias voltage $V_{\mathrm{DS}}=10\;\mathrm{mV}$ was applied and simultaneously the current *I*_DS_ was measured. The metallic heating elements were fed with an AC current injected by employing the sine output a SR830 Lock‐in amplifier. The AC voltage output of the SR830 was connected with a resistor in series with the metallic serpentine, such that R_injection_ > >R_heater_.

### Computational Methods

Atomistic simulations were performed using NAMD software package.^[^ ^29^ ^]^ CHARMM General Force Field^[^ ^30^ ^]^ was used to parameterize PEO, whereas partial charges of ionic species were extracted from ab‐initio MD simulations performed with CP2K^[^ ^31^ ^]^ (see Supporting Information for details). Atomistic trajectories were then used to evaluate the pair distribution functions to calibrate a Coarse Grained Force Field (CG‐FF) based on a single bead per monomer.^[^ ^32^ ^]^ The CG‐FF was parameterized based on a multi‐scale approach previously used for biopolymers^[^ ^33^, ^34^ ^]^ and illustrated in Section S2, Supporting Information. The results of these simulations were employed for the finite element modeling (performed with COMSOL Multiphysics) of the temperature and mass density profiles in the polyelectrolyte as reported in Section S3, Supporting Information.

## Conflict of Interest

The authors declare no conflict of interest.

## Supporting information

Supporting Information

## Data Availability

The data that support the findings of this study are available from the corresponding author upon reasonable request.
