# Supplementary material for: Heat‐Driven Iontronic Nanotransistors
Source: Adv Sci (Weinh). 2023 Jan 25;10(7):2204120. doi: 10.1002/advs.202204120 (PMC9982553; doi:10.1002/advs.202204120)
Supplement: Supplementary file 1 — Supporting Information [file ADVS-10-2204120-s001.pdf]

Heat-Driven Iontronic Nanotransistors

Supporting Information

Domenic Prete[1], Alessia Colosimo[1,2], Valeria Demontis[1], Luca Medda[1], Valentina Zannier[1], Luca Bellucci[1], Valentina Tozzini[1], Lucia Sorba[1], Fabio Beltram[1], Dario Pisignano[2], and Francesco Rossella[3]

[1]NEST, Scuola Normale Superiore and Istituto Nanoscienze-CNR, Piazza San Silvestro 12, 56127, Pisa, Italy
[2]Università di Pisa, Dipartimento di Fisica, Largo Bruno Pontecorvo, 3, 56127, Pisa, Italy
[3] Dipartimento di Scienze Fisiche, Informatiche e Matematiche,
Università di Modena e Reggio Emilia, via Campi 213/a, 41125 Modena, Italy

S. I. Molecular Dynamics Simulations

Multi-scale simulations of the system are performed following the scheme illustrated in Fig S1.

(a) We first define a monomer-based mapping of the atomistic structure to a CG one. Each monomer is described by a single bead for the group $-CH_2-O-CH_2-$ (7 atoms) while the extremal one include only $-CH_2-OH$ (5 atoms) for the neutral end, or $-CH_2-O^-$ (4 atoms) for the charged one. The Na^+ ion is represented atomistically. The CG bead is placed on the center of mass of the group.

(b) Classical atomistic simulations are performed mixing randomly 500 9-mer PEG neutral chains with 500 dissociated PEO-Na chains (500 PEO^- and 500 Na^+). The simulation setup is reported in the panel. The general CHARMM FF was assigned (using CGenFF program) for PEO chains [1],[2]. The partial charges were rescaled of a factor 0.8 with respect to the original partial charges of PEO following a common prescription for ionic liquids[3],[4]. The partial charge of Na+ ions was extracted from a Car-Parrinello MD simulation of a pre-equilibrated system including of 8 PEO and 8 PEO^- Na^+ in a cubic box of 21 $Å^3$. *Ab initio* calculations were performed with the software CP2K[5] using a standard setup[6] with Perdew-Burke-Ernzerhof (PBE) exchange and correlation functional and Grimme's third-generation dispersion corrections (D3). The system was relaxed for 14 ps and the final conformation was used to evaluate the Restrained Electrostatic Potential (RESP) charges[7]. The classical atomistic trajectories are post-processed by coarse graining according and subsequently the distributions of the internal coordinates (bead-bead distances, angles and dihedrals) are built as reference for the parameterization.

(c) The Potentials of Mean Force (PMF) obtained by Boltzmann inversion of the internal variables distribution is then used to parameterize the CG-FF according to a protocol previously used for minimalist models of proteins[8],[9]. The Force Field terms and parameterization finally used is reported in Table S.1. Simulations are performed with the setup reported in the panel and diffusion coefficients of the different components are evaluated. It is to be remarked here that the D reported in the plot accounts for the fictitious acceleration factor due to the coarse graining. This factor is extremely variable depending on the system. In this case it was evaluated to be ~300, by comparing the first relaxation phases of the atomistic systems to that of the CG system. The t* reported in the plot of the diffusion coefficient is renormalized by this factor. Accordingly renormalized D coefficient lie in the experimental range. Panel c also reports a representation of the system with the different components

delimited by isosurfaces of the average densities in corresponding color, evaluated averaging over the last ~100 ns of simulation. The charged component (red and green) appear to form a percolated cluster separated by the neutral one (grey). The cluster formation is confirmed by the snapshots taken at the end of simulation, where chains of alternating Na+ with the polar head of the dissociated PEO are visible. Nevertheless, the ionic components appear to diffuse (see inset of D plot in (c), where the trajectories of a selected molecule for each component is reported). The diffusion mechanism of Na+ appears to occur by hopping along these chains, and therefore is more efficient than the one of the polymeric components. This is compatible with its ~1.4 factor larger mobility of Na+ with respect to the anionic polymeric component.

**Force Field**	$U = \sum_{bonds} u_b(r) + \sum_{ang} u_a(\theta) + \sum_{dih} u_d(\phi) + \sum_{(i>j)\wedge[(i,j)\notin(bond,ang,dih)]} u_{vdw}(r_{ij}) + u_{el}(r_{ij})$		
**Bead types**	**Composition**	**Mass**	**Charge**
**Na**	Na^+	22.9900	+0.44
**EO**	-CH2-O-CH2-	44.0500	0.0
**SP**	-CH2-OH	31.0300	0.0
**SC**	$-CH2-O^-$	30.0300	-0.44
**FF Terms**	**Analytic form**	**parameters**	
$\boldsymbol{u_b(r)}$	$\frac{1}{2}k(r-r_0)^2$	$r_0^{EO-EO} = 3.3$ Å<br>$r_0^{EO-SP,SC} = 2.9$ Å	$k = 15$ kcal/mole Å^2
$\boldsymbol{u_a(\theta)}$	$\frac{1}{2}k_\theta(\cos\theta - \cos\theta_0)^2$	$\theta_0 = 130$ deg	$k_\theta = 20.3$ kcal/mole
$\boldsymbol{u_d(\phi)}$	$\sum_{i=1,4} A_i[1+\cos(m_i\phi - \delta_i)]$	**A$_i$ δ$_i$ i**<br>0.469 180.0 1<br>0.043 0.00 2<br>0.079 0.00 3<br>0.029 0.00 4	
$\boldsymbol{u_{vdw}(r)}$	$4\varepsilon\left[\left(\frac{\sigma}{r}\right)^{12} - \left(\frac{\sigma}{r}\right)^{6}\right]$	**EO EO** 0.3 4.1<br>**EO SP** 0.3 4.1<br>**SP SP** 0.8 3.5<br>**EO SC** 0.3 4.1<br>**SP SC** 2.0 3.0<br>**SC SC** 2.0 3.5<br>**Na Na** 0.046 2.5<br>**Na EO** 3.0 2.4<br>**Na SP** 3.0 2.4<br>**Na SC** 0.17 2.8	
$\boldsymbol{u_{el}(r)}$	Pure Coulomb with effective charges		

Table S.1. Coarse Grained Force Field and parameters.

The diffusion mechanisms are better understood in the Supporting Movies (box.mpg and hop.mpg). In the first one we highlight one molecule per component with respect to the rest of the molecules in the box (shown as

transparent). The larger mobility of Na^+ (in green) is apparent and also visible is the motion occurring by hopping. This is even clearer in the second movie (hop.mpg) where a single Na^+ ion is highlighted in yellow and observed to hop along the chains of alternating Na^+ (green) and polar head O^- (red) of the dissociated PEO (static images from the movies are reported in Fig S.2 for reference to the movies given in separate files).

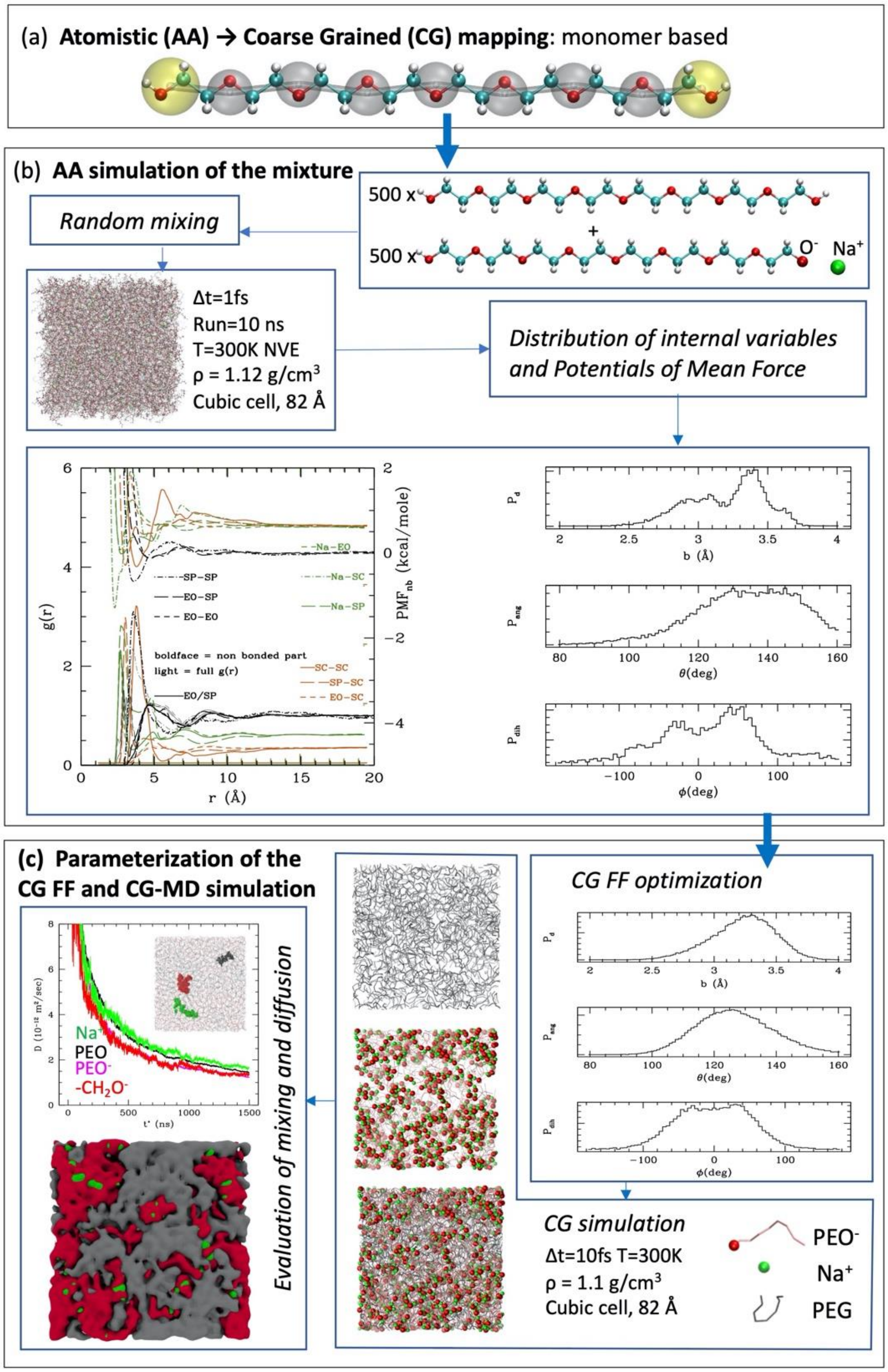

Figure S1: Scheme of the multi-scale simulation. Panel (a): Atomistic to Coarse Grained mapping scheme (b) Flux diagram of the atomistic simulations. Following the arrows: the neutral/charged polymer and sodium mixture is prepared randomly mixing a total of 1000 chains and 500 Na ions in a cubic box of size~8 nm. The system is equilibrated with the parameters given, and

subsequently the distribution of internal variables (bonds, angles, dihedrals and pair distribution functions) are evaluated. Panel (c): the distribution functions evaluated from atomistic simulations are used to optimize the CG-FF and the simulations are performed on a CG system homologous to the atomistic one. The time scale reached are much longer, as shown in the box at the left, and the diffusion coefficient of the different species are evaluated. The box at the left also report a representation of the mixture showing the different components in colors (red for the charged polymer, green for sodium and gray for the neutral polymer), represented as iso-surfaces of density for each component.

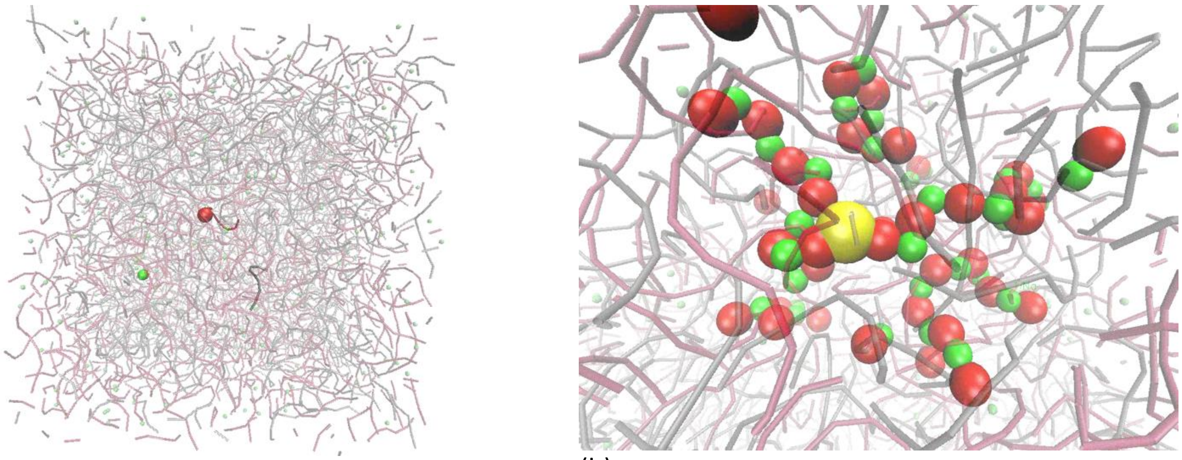

(a) (b)

Figure S2: (a) Static snapshot of the Supporting movie *box.mpg* (from the CG simulation, one molecule for each component is highlighted, others shown as transparent). (b) Static snapshot of the supporting movie hop.mpg, zoom-in of the same simulation with a Na^+ highlighted in yellow, others in green, forming a chain with polar heads of the dissociated PEO chains (in red)

S.II. Control Measurements

S.II.a: FTIR Spectra

After the synthesis of Na functionalized PEO samples as described in the experimental methods of the main text, samples were analyzed by means of Fourier Transform Infrared Spectroscopy in order to determine the success of the functionalization procedure. A typical acquisition is reported in Figure S3, showing that for functionalized samples the O-H peak at $\sim 3500\ cm^{-1}$ disappears completely, consistently with the deprotonation of the terminal CH_2OH group of the polymeric chain.

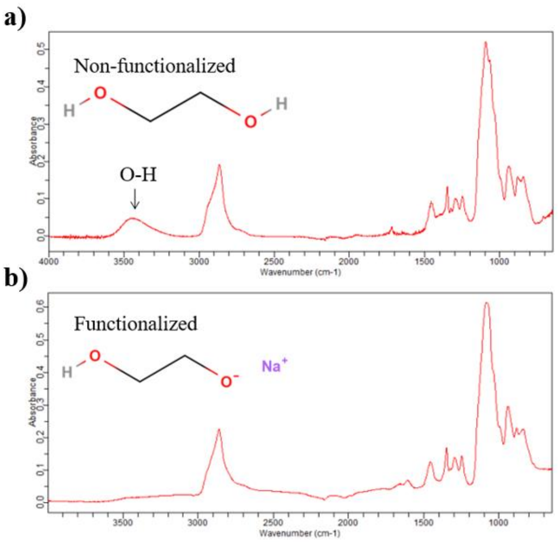

Figure S3: FTIR spectra of PEO sample before (a) and after (b) the sodium treatment. The peak at $\sim 3500\ cm^{-1}$ corresponding to O-H bonds disappears after the functionalization, demonstrating the success of the deprotonation procedure leaving charged CH_2O^- groups at the end of PEO chains, along with free Na^+ ions in the polymeric matrix.

S.II.b: Ion gating operation with Na-functionalized PEO

Prior to thermoelectric gating measurements, we perform conventional ion gating measurements by driving the polyelectrolyte with the application of a DC voltage to a metallic counter-electrode. The achieved field effect modulation of the electrical conductivity of the nanowire, reported in Figure S4, is compatible with the functionalization performed on the polymer and the presence of free ions able to perform ion gating.

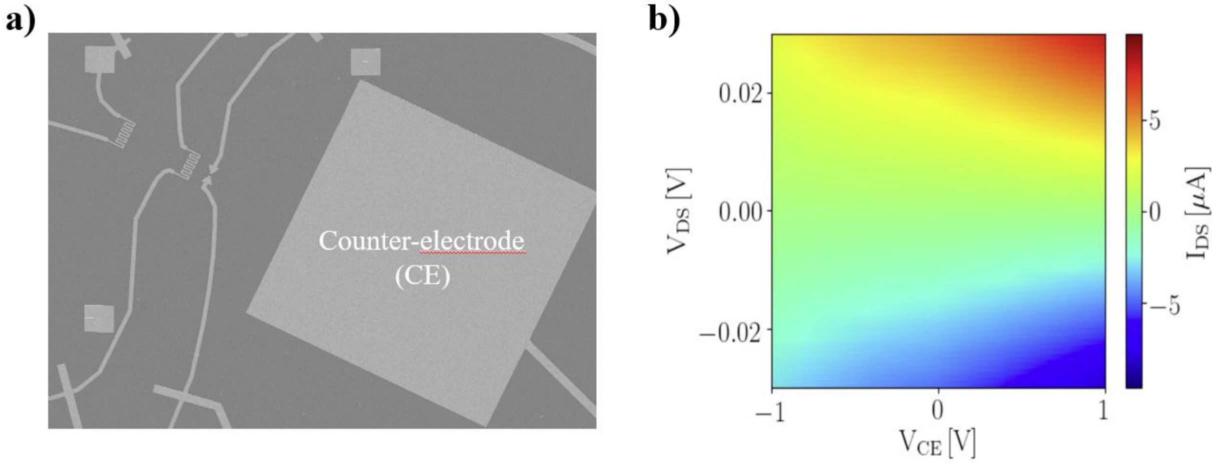

Figure S4: Ion gating device operation with a functionalized PEO droplet. a) Prototypical device fabricated with a square counter-electrode to perform conventional ion gating on the InAs nanowire by employing Na functionalized PEO as the gate dielectric. b) Field effect modulation of the active channel current is observed in such device architecture.

S.II.c: Device operation with non-functionalized polymer

In order to assess the origin of the modulation of the electrical conductivity of the nanowire, device operation was tested when the nanowire is immersed in a droplet of non-functionalized droplet of polyethyleneoxide (molecular weight 400 g/mol). The droplet was treated as an electrolyte gate and a DC voltage bias was applied to a counter-electrode, in order to perform conventional field effect modulation of the electrical transport properties of the nanowire by means of ion gating, if any ion is present natively in the polymer. Figure S5 shows that absolutely no field effect modulation is achieved, clearly due to the absence of any ion in the non-functionalized electrolyte. This means that even if thermally driven, the non-functionalized droplet is not capable of performing field effect on the semiconducting nanowire. Moreover, we also fed the heating elements similarly to what is reported in the main text, finding out that due to the absence of the ions in the droplet no relevant modulation of I_{DS} is achieved.

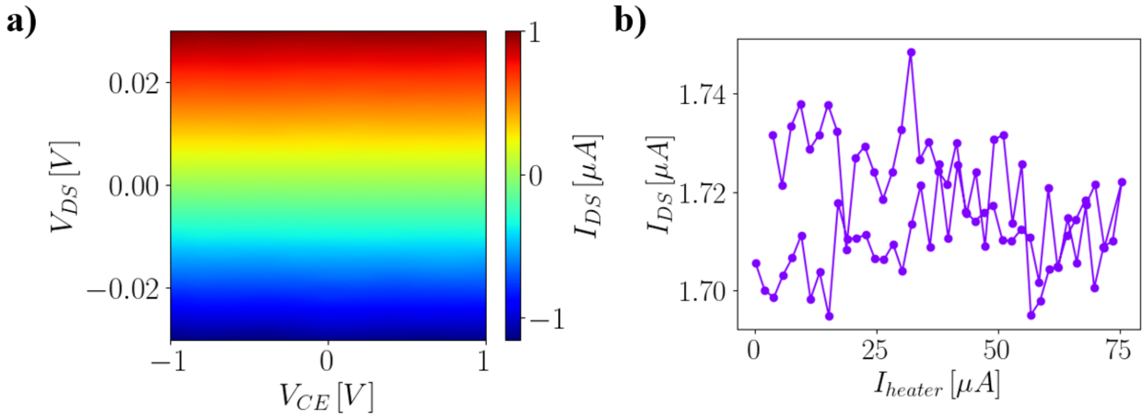

Figure S5: Electrostatic device operation with a non-functionalized PEO400 droplet. (a) A DC voltage is applied to a metallic counter-electrode, similarly to what is done with electrolytes for conventional electrostatic ion gating. No field effect modulation of the electrical conductivity is achieved, in accordance with the absence of ions in the employed droplet. (b) The heating element is fed with an AC current similarly to what is performed in the measurements reported in the main text. No relevant modulation of the channel current is achieved.

S.II.d: Device operation without polyelectrolyte

In order to exclude any spurious contribution on the modulation of the electrical conductivity of the nanowire not coming from the polyelectrolyte, we have tested device operation without applying any droplet on the device. We have injected the heating current through the metallic heaters and measured the current flowing in the nanowire, similarly to what is reported in the main text for thermoelectric gating experiments. In this case, if the heating of the substrate is such that an abrupt change in temperature in caused locally on the nanowire, its resistance may vary due to the dependence of the electrical resistance on temperature. As clearly visible in Figure S6, this is not the case, confirming that the observed modulation of the nanowire electrical conductivity is caused by the accumulation of sodium cations rather than any thermal effect unrelated to the employed polyelectrolyte.

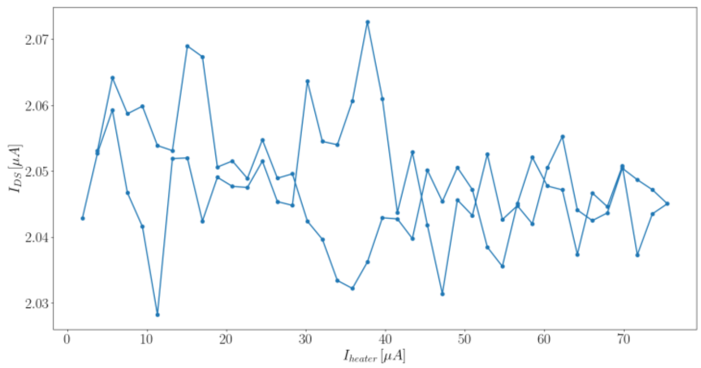

Figure S6: Current flowing through the nanowire when the heating element is fed, and no droplet is casted on the device. The temperature variation due to the substrate heating is not relevant for the modulation of the resistance of the nanowire.

S. III. Finite Element Modeling

The finite element analysis of the system has been performed with COMSOL Multiphysics suite. Local temperature at the heater location is computed by calculating the electric field and current density in the metallic serpentine, and used as heat source for the heat equation in the polyelectrolyte droplet:

$$\nabla \cdot (\kappa \nabla T) = \boldsymbol{J} \cdot \boldsymbol{E},$$

Where:

$$\nabla \cdot \boldsymbol{J} = Q_j$$

$$\boldsymbol{J} = \sigma \boldsymbol{E}$$

$$\boldsymbol{E} = -\nabla V$$

Moreover, the developed model computes the mass transport of a three-component mixture by solving the following equation:

$$\left(\rho \frac{\partial \omega_i}{\partial t}\right) + \nabla \cdot \boldsymbol{j} = 0,$$

Where $\boldsymbol{j}$ is the mass density of current of species i, ω_i is the mass fraction of the species i and:

$$\boldsymbol{j} = -\left(\rho D_j^f \nabla \omega_j + \rho \omega_j D_j^f \frac{\nabla \mathrm{M_n}}{M_n} - \boldsymbol{j}_{c,j} + D_j^T \frac{\nabla T}{T}\right),$$

$\boldsymbol{j}_{c,j} = \rho \omega_j \sum_k \frac{M_j}{M_n} D_k^f \nabla \mathrm{x_k}$, D_k^f being Diffusion coefficient of species k , $M_n = \left(\sum_j \frac{\omega_j}{M_j}\right)^{-1}$, M_j being molar mass of species j and $x_k = \frac{\omega_k}{M_k} M$, $\frac{1}{M} = \sum_{i=1} \frac{\omega_i}{M_i}$. The variables the model solves the equation for are the voltage in the heater, V, and the mass fraction of each component of the mixture, $\omega_j, j = 1,2,3$.

The model can be used to compute the spatial distribution of mass, charge, and temperature for specific operational configurations determined by heating current amplitude and frequency and waiting time between current application and system measurement, meaning that the simulation lets the system evolve in time for a time interval equal to the experimentally waited time between measurements.

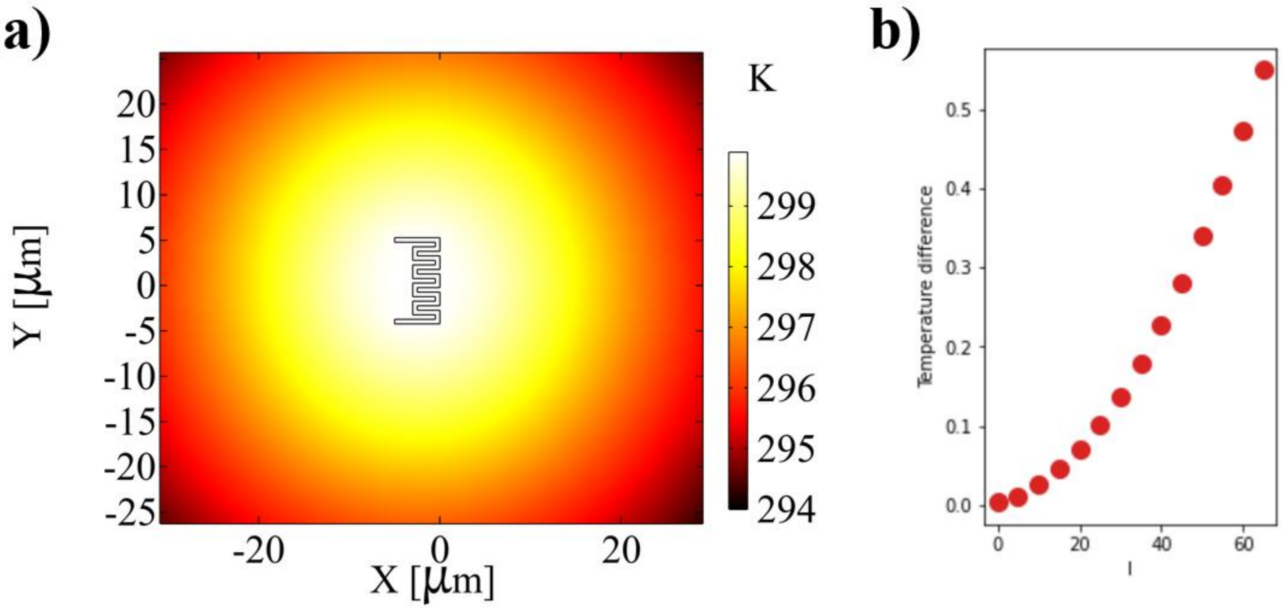

Figure S7*:* (a) Computed temperature profile for $I_{heater} = 75\ \mu A$. (b) Temperature difference between the points located at $(x_0, y_0) = (0\ \mu m, 0\ \mu m)$ and $(x_1, y_1) = (7\ \mu m, 0\ \mu m)$ sweeping the heater feeding current as explained in the main text.

References

[1] K. Vanommeslaeghe and A. D. MacKerell, Jr.* *Automation of the CHARMM General Force Field (CGenFF) I: Bond Perception and Atom Typing J. Chem. Inf. Model.* 2012, 52, 12, 3144–3154 (2012)
[2] K. Vanommeslaeghe, E. Prabhu Raman, A. D. MacKerell, Jr Automation of the CHARMM General Force Field (CGenFF) II: Assignment of Bonded Parameters and Partial Atomic Charges *J. Chem. Inf. Model.* 2012, 52, 12, 3155–3168 (2012)
[3] Quantitative Prediction of Physical Properties of Imidazolium Based Room Temperature Ionic Liquids through Determination of Condensed Phase Site Charges: A Refined Force Field Anirban Mondal and Sundaram Balasubramanian* *J. Phys. Chem. B* 2014, 118, 12, 3409–3422 (2014)
[4] Revisiting OPLS Force Field Parameters for Ionic Liquid Simulations
Brian Doherty, Xiang Zhong, Symon Gathiaka, Bin Li, Orlando Acevedo, *J. Chem. Theory Comput.* 2017, 13, 12, 6131–6145 (2017)
[5] T D Khune et al, CP2K: An electronic structure and molecular dynamics software package - Quickstep: Efficient and accurate electronic structure calculations J. Chem. Phys. **152**, 194103 (2020)
[6] Water splitting of hydrogen chemisorbed in graphene oxide dynamically evolves into a graphane lattice
L Ciammaruchi· L Bellucci, G Comerón, C G Martínez, D Sánchez, Q Liu, V Tozzini, J Martorell Carbon 153, , Pages 234-241 (2019)
[7] C Campañá, B Mussard, T K. Woo Electrostatic Potential Derived Atomic Charges for Periodic Systems Using a Modified Error Functional **J. Chem. Theory Comput.* 2009, 5, 10, 2866–2878 (2009)
[8] A Di Fenza, W Rocchia, V Tozzini *Complexes of HIV-1 integrase with HAT proteins: Multiscale models, dynamics, and hypotheses on allosteric sites of inhibition* Proteins: Structure, Function, and Bioinformatics 76 (4), 946-958 (2009)
[9] F Delfino, Y Porozov, E Stepanov, G Tamazian, V Tozzini *Evolutionary Switches Structural Transitions via Coarse-Grained Models* Journal of Computational Biology 27 (2), 189-199 (2020)
